# Neuroticism related differences in working memory tasks

**DOI:** 10.1371/journal.pone.0208248

**Published:** 2018-12-17

**Authors:** Rahmi Saylik, Andre J. Szameitat, Survjit Cheeta

**Affiliations:** 1 Medical Science Division, Department of Experimental Psychology, University of Oxford, Oxford, United Kingdom; 2 Division of Psychology, Department of Life Sciences, Centre for Cognitive Neuroscience (CCN), Brunel University London, Uxbridge, United Kingdom; Universty of Sydney, AUSTRALIA

## Abstract

Two influential theories relating to personality traits, i.e. arousal-based theory (ABT) and attentional control theory (ACT), made predictions on how neuroticism may affect task performance. ABT suggested that high neurotics perform worse than low neurotics in all difficult tasks, whereas they perform similar in easy tasks. On the other hand, ACT suggested that high neurotics perform worse than low neurotics only if the task relies on central executive functions of working memory (WM), such as switching or inhibition. However, currently it is still unclear whether neuroticism affects all difficult tasks, as proposed by ABT, or whether it is specific to certain tasks, as proposed by ACT. To test this, we used the Cambridge Neuropsychological Tasks Automated Battery (CANTAB) as our test tool and we selected three working memory tasks which tested the effect of neuroticism on both the central executive system (CES) and the WM storage system (i.e. visuospatial sketchpad) in 21 low and 24 high neurotics. Results showed that high neurotics, as compared to low neurotics, exhibited lower performance only when the working memory task is specifically associated with switching and/or inhibition, but not in a task which is associated with the visuospatial sketchpad. We conclude that the results support the ACT rather than the ABT, because high levels of neuroticism impaired behavioural performance specifically in demanding tasks associated with switching and inhibition, but not in tasks associated with the visuospatial sketchpad.

## Introduction

Neuroticism is a personality trait that refers to a constant inclination towards negative emotions and higher levels of anxiety [1,2]. This results in increased arousal and worry related thoughts, which impair cognitive processing [3,4]. Highly similar or even identical concepts rooted in different models of personality have been termed trait anxiety and negative affectivity [5–7].

One of the key cognitive functions in humans is working memory, because it is involved in memory, the control of attentional resources, conflict resolution, task switching, decision making, planning, and monitoring [8–11]. Due to this importance, we aimed to investigate the effects of neuroticism on different aspects of working memory (WM). One of the most widely used models of WM was suggested by Baddeley and Hitch (1974). In this model, WM is conceptualised as consisting of two basic short-term stores (the phonological loop and the visuospatial sketchpad), and a central executive system [12,13]. The phonological loop and visuospatial sketchpad temporarily store auditory and visuospatial information, respectively [12,13]. The central executive system (CES) supervises these memory stores and in addition can manipulate information and control attention [12,13]. In that model, it has been proposed that the CES might to some degree have divisible functions rather that being an unitary system [8–11]. Following Baddeley and Hitch’s model Miyake et al. (2000) conducted an impressive study investigating the function of CES. Miyake et al, (2000) used latent variable analysis to determine the main functions of the central executive system. He selected several standard WM tasks that have been proposed for investigating central executive functions by various researchers (Baddeley, 1996a; Smith & Jonides, 1999). 137 participants performed several tasks including the Wisconsin Card Sorting Test (WCST), and the Tower of Hanoi (TOH) task. This study suggested that there are three main functions of the CES that can be distinguished but also correlate with each other. These functions of the CES can be defined as shifting, inhibition and updating [14,15]. Inhibition refers to the suppression of task irrelevant stimuli that can potentially cause interference [16,17]. Switching refers to flexibility in shifting attention between two tasks, operations or mental sets [18]. Updating refers to refreshing and monitoring of mental representations during the processing of tasks [15,18].

In regard to the effects of neuroticism on WM, M.W. Eysenck, Derakshan, Santos, & Calvo, (2007) proposed the attentional control theory (ACT), which is based on the above described Baddeley & Hitch’s (1974) working memory (WM) model. ACT proposes that when tasks become difficult/complex and stressful, high levels of neuroticism cause high levels of worry-related arousal [18]. Such arousal causes task-irrelevant mental activities such as rumination or worry related thoughts [17–19]. Therefore, high neurotics do not only have to deal with the mental activities related to processing the task, but in addition with the task-irrelevant activities [17–19]. ACT proposes that this situation specifically impairs the inhibition, switching and updating functions of the CES, while other CES functions (such as planning) and the storage systems (phonological loop, visuospatial sketchpad) remain largely unaffected [17–19]. This is because these three CES functions would impose the highest demands on sustained attention for efficient task processing and because task-irrelevant mental activities would limit the investment of mental effort into these functions [17–19].

It should be noted that ACT suggests that in some cases (e.g. when stress is induced or using threatening stimuli) neuroticism impairs updating as well [19]. However, this impairment is less evident in situations without stress induction because although being a function of the CES, updating also involves the storage systems, which are not affected by neuroticism [17,19]. Due to this vague status, we did not investigate updating in the current study.

While ACT makes specific predictions about the WM functions which are impaired by high levels of neuroticism, the arousal-based theory (ABT) of H.J. Eysenck (1967) suggests that high neurotics generally perform worse than low neurotics in any kind of difficult or complex task. In detail, the theory of H.J. Eysenck (1967) is based on the well-known finding that arousal and performance are linked by an inverted U-shaped function, i.e. if the task is easy (very low arousal) or extremely difficult/complex (very high arousal), performance is low, while an intermediate level of arousal results in highest performance. H.J. Eysenck (1967) suggested that at low and high levels of arousal, low and high neurotics actually show the same performance, one of the explanations being floor and ceiling effects, respectively. However, H.J. Eysenck (1967) suggested that high neuroticism results in a lower arousal activation threshold so that the inverted U-shaped function is skewed to the left, i.e. in the direction of lower arousal [1,4]. As a consequence, high neurotics reach the peak of perfect performance already at much lower arousal levels and show decreasing performance already at difficult/complex tasks. In other words, low and high neurotics are expected to differ in their performance of difficult/complex tasks that causes higher arousal level in high neurotics [1,4]. Importantly, this effect is independent of the source of difficulty/complexity, i.e. it holds for all tasks and not only for some CES functions as proposed in the ACT. Consequently, in contrast to ACT, the ABT predicts that task difficulty/complexity always results in higher arousal and task-irrelevant activities, which impair any ongoing task-relevant activities, irrespective of their nature [1,4]. The current study aimed to test the effects of high levels of neuroticism on different functions of WM to first differentiate on a broader level between ACT and the ABT and, second, to investigate whether the specific ACT predictions about the affected WM functions can be confirmed. It is important to find out whether the detrimental effect of neuroticism is task specific or general because a considerable number of studies have been performed based on these theories. Thus this investigation will help to understand whether recent assumptions of theoretical accounts are correct and allow reaching deep insights of potential causes in cognitive impairments due to neuroticism. There are some other related theoretical accounts which are in line with ACT when assessing potential reasons of detrimental effect of neuroticism such as dual mechanism of control [20], task-person-situation[21] and the five factor theory of personality. For instance, in ACT, it has been commonly suggested that low level neuroticism involves the top-down goal-driven system, which is involved in sustained representation of cognitive task-relevant goals [19]. Conversely, high levels of neuroticism are associated with the bottom-up goal-directed system. In this system, transient representations of stimulus related activities are affected due to disruption of worry related thoughts during task processing [19]. Therefore, high levels of neuroticism lead to worry related thoughts which limit investment of mental resources into the task [19]. A very similar assumption holds in the dual mechanism control theory. This theory suggests this while using different terms: reactive (bottom-up) and proactive controls (top-down) [20]. Furthermore, evidence suggests that intrinsic motivation plays a pivotal role in enhancing the highest levels of motivation and performance which is linked to the top-down goal-directed system. Individuals with emotional stability (low neuroticism) have intrinsic motivation, therefore, they are more focused and goal directed when performing a task [21]. In addition, according to the five-factor theory of personality, neuroticism related traits are associated with reduced function of the serotonergic system which disturbs attentional control[22].

Previous evidence has confirmed that high levels of neuroticism indeed affect predominantly difficult tasks demanding the CES [3,23–28]. For instance, Szameitat, Saylik & Parton (2016) were able to show that, when compared to low neurotics, high neurotics are specifically impaired in multitasking, but not when performing a single task. This study also supported the idea that high neurotics invest less mental resources into the task. They showed that brain activation in the lateral-prefrontal cortex, which was specific to multitasking, was lower in high than in low neurotics [29]. Several studies used other paradigms, such as the n-back task [30], the go-no-go task [31], the delayed-response working memory task [32], and the probe task [33,34] to investigate neuroticism. However, these studies were not able to differentiate whether high neurotics are affected in all difficult tasks or only in some specific ones, nor were they able to assess whether the impairments are as specific as predicted by the ACT.

In the present study, we used the Eysenck Personality Questionnaire (1975) (EPQ) to create two extreme groups (low and high neurotics) and compared the performance of these two groups in standard WM tasks. ACT proposed that in particular the CES functions inhibition and switching, are impaired. To test this, we selected tests based on the study of Miyake et al. (2000) who provided a highly detailed description of how to assess CES functions. In more detail, we used the CANTAB as our test tool and chose the Intra-Extra Dimensional Shift task (IED; similar to the Wisconsin Card Sorting Test, WCST) for testing the effect of neuroticism on switching and inhibition. Furthermore, to test the effect of neuroticism on planning we chose the Stockings of Cambridge test (SOC; similar to the Tower of Hanoi task). In addition, we selected the spatial working memory task (SWM; similar to the Corsi Block task, assessing the visuospatial store) because we wanted to test whether neuroticism specifically affects the main CES functions only, and not the storage systems. We chose a task assessing the visuospatial Sketchpad (VSSP) instead of the phonological loop (PL) because it has been suggested that the PL function might be affected by the level of neuroticism [19] because of task-irrelevant mental activities such as rumination and the involvement of the PL in inner speech, To manipulate task difficulty, we used different memory loads in the SWM task and different number of moves in the SOC task.

It should be noted that there is no WM task that measures only a single WM function, because most tasks are tapping into multiple WM functions at the same time [14,15]. However, the magnitude of a function can be greater than the other functions in a WM task. Therefore, in the current study, each task was selected because it predominately involved the respective WM function [14,15]. Taken together, the first aim of the current study was to differentiate between ACT and ABT by particularly comparing performance in the difficult SWM task. While the ACT predicts comparable performance in this task for high and low neurotics, the ABT predicts poorer performance for high neurotics. The second aim was to test the ACT in more detail by assessing whether the proposed CES sub-functions of inhibition and switching are impaired by high levels of neuroticism.

## Methods

### Participants

To create extreme groups of high and low neurotics (High-N and Low-N, respectively), we screened 400 participants using the 24-item neuroticism scale of the Eysenck Personality Questionnaire at Brunel University campus [35]. Five participants were excluded because of current or previous depression or anxiety disorders according to the history of past or current psychiatric or neurologic disorders questionnaire. From the people screened using the neuroticism scale of EPQ, 45 people were selected to take part in the final experiment: 24 (12 female) were in the High-N group (mean EPQ score = 18.10, range = 16–24) and 21 (8 female) were in the Low-N group (mean EPQ score = 3.52, range = 1–6). The two groups were roughly matched for age (High-N = 21.21 and Low-N = 22.86) and gender (High-N: 50% female, Low-N: = 40%) and predicted IQ (High N mean NART score = 104, and Low N mean NART score = 106) i.e. scores based on National Adult Reading Test II (NART II) showed that predicted IQ was higher than 70 in all participants [22] and high and low neurotic participants did not differ significantly regarding predicted IQ level based on independent t-test. All of the participants were Brunel University students, right-handed as assessed by the Edinburgh Inventory [36] and had normal or corrected to normal vision. Before participation each participant gave written informed consent. The participants were paid £10 for participating for one hour. The study was approved by the Department of Life Sciences ethics committee at Brunel University.

### Materials

We used the 21-item Neuroticism scale of the EPQ to assess Neuroticism [35]. Participants were classified as High-N when they scored over 16 and as Low-N when they scored below 6 [37–39]. To avoid potential confounding effects from depression, participants with a BDI (Beck Depression Inventory) [40] score of 15 or higher were excluded. A self-designed questionnaire was used to exclude participants who had a history of psychiatric or neurological illness. Also, an alcohol and caffeine consumption survey was used to exclude possible effects of alcohol and caffeine. No participant was colour blind as tested by the Ishihara colour blindness test [41]. Finally, we used National Adult Reading Test II (NART II) to avoid potential confounding effects due to predicted IQ. Therefore, participants who scored lower than 70 in this test were excluded [22].

#### Cognitive stimuli: Cambridge Neuropsychological Tasks Automated Battery (CANTAB)

The current study included three CANTAB (http://www.cambridgecognition.com/cantab/) tasks, which were: (a) Stoking of Cambridge (SOC), (b) Spatial Working Memory task (SWM); (c) Intra-Extra Dimensional Shift task (IED). The administered tasks are described briefly below.

#### Stockings of Cambridge (SOC)

The SOC is closely related to the Tower of London task and is designed as a spatial planning task that is also suggested to be associated with the inhibition function. In this task, two displays are presented on a screen, one located at the top of the computer screen and the other one at the bottom. Each display contains three coloured balls. The participants are required to look at the top pattern (configuration) and copy that pattern in the bottom pattern by moving the coloured balls to their proper location on the touch screen (make the bottom pattern same as the top pattern). The participants touch the required balls and then touch the position of where the ball should be moved to. The patterns to be copied vary in their complexity, requiring between at least 2 (low difficulty) and 5 (high difficulty) moves. Two dependent variables which are ‘the number of mean moves’ and ‘minimum moves’ were used. The number of mean moves refers to the mean number of moves that have been done by the participants to complete a test problem for each level of complexity [42,43]. Thus, a participant who had a higher mean number of moves has been less successful than one who had a smaller mean number of moves [42]. In addition, the total number of minimum moves refers to the test problems which are perfectly completed by the fewest possible number of moves across all levels [42].

#### Spatial/visual working memory task (SWM)

The SWM is associated with the Visuospatial Sketchpad (VSSP) component of working memory. In this task, participants were presented a spatial array which included coloured boxes and an empty column next to the array. At the beginning of the task, participants were asked to find hidden yellow tokens in a spatial array of four, six, or eight coloured boxes on the screen. For this, participants had to tap on a box (using a touchscreen) to reveal whether it contained the hidden yellow token or not. When they found a token in a box, the token moved to the column on the right and then participants had to visit the other boxes to find another token. Participants were informed that boxes where they found a token before will not hold a token again in the same trial and thus they should seek tokens in the remaining boxes where tokens haven’t been found yet. However, after a token had been found, tokens could appear in boxes which have been visited before, but which were non-targets at that time. Thus, for an efficient strategy, participants needed to keep track of all the boxes where tokens had been found before. Difficulty of the task increased sequentially by showing 4 boxes in the first trial, 6 in the second, and 8 boxes in the last trial. We assessed the error rates of visuospatial working memory separate for 4, 6, and 8 boxes and, in addition, the sum of those as a measure of total number of errors [44]. In detail, average of errors for each trial which are 4, 6, and 8 boxes were calculated separately and then average of total errors for all trials was calculated.

#### Intra-extra dimensional shift task (IED)

IED is an attentional set shifting task, an analogue to a computerized version of the Wisconsin card sorting test [45], and it is used to measure the switching and inhibition functions of the central executive system [42]. In this task, participants have to identify rules which determine the correct stimulus among up to four potential stimuli. They have to avoid perseveration which refers to sticking to the old rule during rule alterations. In more detail, there are two categories in this task. The first category is intra dimensional shift which consisted of either two coloured shapes or two ramified lines. For instance, the two-coloured shapes, one on the left and one on the right side on the screen, are always shown at the same time, and there are several rules which determine which coloured shape is the correct one. Participants have to use the feedback they receive after each response (correct / wrong) to find out the current rule. Once they have found the rule and responded correctly for six times, the rule is changed without notification of the participant, who again has to learn the new rule by using the feedback. Likewise, two ramified lines could be presented one on the left and one on the right side on the screen and participants must perform as in the shapes task. In the dual version of task (coloured shapes and two ramified lines) an intra- dimensional shift is required whenever the rule changes whether within colour, or within ramified lines. The other category is called extra dimensional shift which consisted of compounded set of shapes and ramified lines; one compound stimuli on the right and the other on the left. In more detail, participants always see four objects on the screen, a compounded set of a coloured shape and a white line on the left, and a compounded set of a coloured shape and a white line on the right, and participants have to just indicate whether the left or right is correct. Participants perform the same rule-learning task across objects categories, but this time rule changes from coloured objects to ramified lines, or from ramified lines to coloured objects. Thus, an extra-dimensional shift is required whenever the rule changes based on the either shapes or lines. It has been shown that the extra-dimensional shifts are more difficult regarding the switching and inhibition functions [46], which is why we focussed on them.

### Procedure

After giving written informed consent, participants were tested individually. First, the participants were given a medical questionnaire and a caffeine consumption survey, the Ishihara colour test and the BDI. Based on these questionnaires, we employed the following exclusion criteria: presence of any past or current major medical, neurological or psychiatric illness that might have diminished cognitive functioning; use of psychoactive medication; consumption of alcohol within the last 24 hours.; consumption of ≥8 cups or ≥ 900mg caffeine within the last 24 hours; scoring over 15 in BDI. Next, participants filled out the EPQ. After this, the participants were seated in front of the CANTAB computer equipped with a 10 ½ in. touch-screen monitor. The SOC, SWM and IED tasks were presented to participants in a counterbalanced order.

The CANTAB tasks were practiced by the participants just before the experiment started because we wanted to introduce the participants to the touch screen and eliminate sensorimotor or comprehension difficulties that might restrict collecting valid data from the participants. Thus, in the practice session, which included half the number of trials, participants practiced all tasks. After the participants had completed the practice, they continued to the study session which involved the three main tasks: the SOC, IED and SWM. The participants were instructed verbally from a script. At the end of the study, participants were debriefed.

## Results

We calculated independent-samples t-tests for each outcome measure in each task with neuroticism as a between group factor. To account for a potentially inflated alpha-error, we used Bonferroni correction for multiple testing within each task. For a family-wise error rate of *p* < 0.05, the Bonferroni corrected thresholds for individual tests in the IED and SWM (4 tests each) are *p* < .0125, and for the SOC (5 tests) *p* < 0.01. In addition, a mixed ANOVA was performed to find out interaction between Neuroticism and manipulated task difficulty in SWM and SOC tasks.

### IED set shifting task

The IED set shifting task strongly relies on the switching and inhibition functions of the CES. ACT as well as the ABT both predict that high levels of neuroticism impair performance in this task. The results demonstrated that indeed high neurotics showed significantly poorer performance than low neurotics. In more detail, the high neurotics made more errors than the low neurotics during the processing of the extra-dimensional set shifting (Table 1), (EDS errors, *t* (43) = 9.25; *p* < .001). Furthermore, the high neurotics had a higher total number of errors across the stages (total errors, *t* (43) = 8.40; *p* < .001). In addition, the high neurotics needed considerably more trials to achieve the stages as compared to the low neurotics (total trials, *t* (43) = 8.93; *p* < .001). Finally, low neurotics completed significantly more stages than high neurotics (stages completed, *t* (43) = 7.36; *p* < .001). Taken together, these results show that high levels of neuroticism result in impaired performance in the IED set shifting task, supporting the hypothesis that neuroticism negatively affects the CES functions of inhibition and switching.

**Table 1:** Number of errors and successfully completed stages for participants with high levels of neuroticism (High-N) and low levels of neuroticism (Low-N).

Outcome Measure	Group	N	Mean	SD	t-test
EDS errors	HIGH N	24	12	10.16	*t* (43) = 9.25, *p* < .001
	LOW N	21	3.52	4.34	
IED stages completed	HIGH N	24	8.54	.83	*t* (43) = 7.36; *p* < .001)
	LOW N	21	9.00	.00	
IED total errors	HIGH N	24	18.38	11.45	*t* (43) = 8.40; *p* < .001)
	LOW N	21	10.76	5.66	
IED total trials	HIGH N	24	81.88	18.18	*t* (43) = 8.93; *p* < .001).
	LOW N	21	69.71	13.85	

### Stockings of Cambridge (SoC)

With respect to CES functions, the SoC relies mostly on planning, and potentially on some inhibition as well (see Discussion section for more detail). Because ACT proposes that neuroticism has no major impact on planning abilities, it predicts that high and low neurotics will perform comparably in all difficulty levels of the SoC. The arousal-based theory, on the other hand, would predict that neuroticism does affect SoC performance, at least in the more difficult conditions with 4 and 5 moves. Our results are in line with the ACT model: High and low neurotics showed highly comparable performance in this task at all levels of difficulty, and consequently statistical tests were non-significant (Tab 2). In more detail, high and low neurotics did not differ significantly with regard to ‘mean number of moves’ and ‘minimum moves’ (all *t* (43) < 1.34, all *p* > .18). Thus, our results show that neuroticism has no or only negligible effects on the CES function of planning, even in difficult tasks, thus supporting the ACT model.

In addition, we calculated a 2x4 factorial ANOVA with the within-subject factor SOC task variables (2 vs. 3 vs. 4 vs. 5 moves) and the between subject factor group (High N vs Low N). The results show that on average the high and low neurotics did not significantly differ on the task performance; *F* (1, 43) = 2.10; *p* = .17] (Table 2). Finally, the difference between high and low neurotics did not significantly change as task difficulty increased, as is evident by the non-significant interaction between the group and task variables [*F* (1, 43) = 2.16; *p* = .16].

**Table 2:** Number of moves needed to complete a test for each level of complexity and mean of the minimum successful moves which are efficiently done across all levels.

Outcome Measure	Group	N	Mean	SD	t-test
SOC Mean 2move	HIGH N	24	2.08	.28	*t* (43) = .437, *p* = .64
	LOW N	21	2.05	.22	
SOC Mean 3move	HIGH N	24	3.12	.33	*t* (43) = .593, *p* = .53
	LOW N	21	3.07	.18	
SOC Mean 4move	HIGH N	24	5.21	.98	*t (*43) = 1.13, *p* = .26
	LOW N	21	4.95	.55	
SOC Mean 5move	HIGH N	24	6.56	1.73	*t* (43) = 1.26, *p* = .23
	LOW N	21	5.98	1.15	
Min. total moves	HIGH N	24	9.20	2.28	*t* (43) = 1.24, *p* = .18
	LOW N	21	10.00	1.41	

#### Spatial working memory (SWM)

Finally, the SWM task strongly relies on the VSSP, but not on the CES. ACT suggests that neuroticism does not affect VSSP performance. The arousal-based theory, however, again suggests that neuroticism affects performance in any difficult task, and therefore predicts that high neurotics should perform worse than low neurotics in the SWM task, at least in the more difficult conditions with 6 and 8 boxes. Our results again support the ACT. In all outcome measures, there was no statistically significant difference between high and low neurotics (all *t* (43) < 1.61, all *p* >.16) (Table 3).

**Table 3:** Mean of error rates across SWM task variables for participants with high levels of neuroticism (High-N) and low levels of neuroticism (Low-N).

Outcome Measure	Group	N	Mean	SD	t-test
SWM total errors	HIGH N	24	19.06	12.58	*t* (43) = .72, *p* = .52
	LOW N	21	17.00	17.91	
4 boxes search	HIGH N	24	.58	1.50	*t* (43) = .43, *p* = .60
	LOW N	21	.81	1.96	
6 boxes search	HIGH N	24	3.21	3.61	*t* (43) = .43, *p* = .66
	LOW N	21	4.57	5.94	
8 boxes search	HIGH N	24	15.04	8.87	*t* (43) = 1.61, *p* = .16
	LOW N	21	11.24	11.03	

In addition, we calculated a 2x3 factorial ANOVA with the within-subject factor SWM task variables (4 vs. 6 vs. 8 boxes) and the between subject factor group (High N vs Low N). The results show that on average the high and low neurotics did not significantly differ on task performance; *F* (1, 43) = .58; *p* = .45]. Finally, the difference between high and low neurotics did not significantly change as task difficulty increased, as is evident by the non-significant interaction between the group and task variables [*F* (1, 43) = 2.24; *p* = .13].

## Discussion

We found that high neurotics had significantly lower performance than low neurotics in all outcome measures of the IED set shifting task. However, high and low neurotics showed highly similar performance without any statistically significant differences in all outcome measures of the SWM and SOC tasks.

The first aim of the current study was to test whether high levels of neuroticism impair performance in all difficult tasks, as predicted by the arousal-based theory (ABT) of H.J. Eysenck (1967), or only in some specific tasks, as predicted by the attentional control theory (ACT) [19]. First, we observed that high and low neurotics differed in the IED. This is in line with previous evidence [3,23–28] and confirms that we indeed created extreme groups which show differences in behavioural performance [37]. However, both theories, ABT and ACT, predict lower performance of high neurotics in IED. Importantly, the two theories make different predictions for the other two tested tasks. While ABT predicts that high neurotics perform poorer in all difficult tasks, i.e. including SWM and SOC [4], ACT predicts that the impairments of high neurotics are more specific and that SWM and SOC should actually be not impaired in high neurotics [19]. The results demonstrated that despite the increasing task demands in the SWM and SOC tasks, the high and low neurotics did not differ in terms of their performance. In particular, even in the more difficult conditions, numerical differences in the measures for high and low neurotics never reached statistical significance. Thus, the results confirm the predictions of ACT, while they are incongruent with ABT.

Our findings are generally in line with previous findings. For instance, M.W. Eysenck, Payne & Derakshan (2005) tested, very similar to the current study, whether neuroticism affects only the CES or also the VSSP, in particular when task difficulty increases in a dual-task situation. In more detail, they showed that if one of the two tasks forming the dual task involved the central executive system (i.e. dual task A: Corsi block task and WCST), high neurotics showed higher task impairment compared to a dual task consisting of two tasks that are associated with the storage systems (i.e. dual task B: Corsi block and articulatory suppression). This is in line with our conclusion that neuroticism affects the CES, but not the VSSP. Furthermore, Eysenck et al., (2005) increased the difficulty of the Corsi block task in both dual tasks (A and B), which affected low and high neurotics similarly. However, an increase in the difficulty of the WCST affected high neurotics more than low neurotics, again illustrating that neuroticism affects only the CES but not the VSSP.

The SOC task results are also consistent with findings which illustrate that high and low neurotics have a comparable performance. For instance, Chan, Goodwin & Harmer, (2007) investigated differences between high and low neurotics using the Tower of London task, which is closely related to the current SOC task. They found that high and low neurotics did not differ in the response times in the Tower of London task. These findings confirm our conclusion that only some CES functions are affected by neuroticism, while for instance the CES function of planning seems to be not affected, or at least only to a much lesser degree.

Finally, the SWM results are in line with findings which showed that increasing task demands in the visuospatial component of WM is not affected by anxiety (with anxiety being closely related to neuroticism) [47]. For instance, Walkenhorst & Crowe, (2009) conducted a series of experiments that included tasks related to the VSSP (e.g. Spatial Span Forwards and Visual Patterns Tasks) with high and low trait anxiety groups. They found that high and low trait anxiety groups did not significantly differ on VSSP tasks in the study [48]. This supports our conclusion that indeed the VSSP is not affected by neuroticism.

H. J. Eysenck (1967) suggested that high neurotics perform worse in difficult tasks than low neurotics, while neuroticism should not affect performance in very easy or extremely difficult tasks. This is because for both high and low neurotics, the arousal level remains on a low level in easy tasks and in extremely difficult tasks the arousal level considerably increases in both groups. Our results demonstrate that this assumption cannot be generalized to the SWM and SOC tasks, because although we varied the task demands in these tasks across a wide range, the high and low neurotics did not differ significantly in their performance. We believe that some of our conditions should have had a difficulty level where, if the ABT would be true, differences between the groups should have been evident. However, in our study, in both tasks high and low neurotics did not differ for any difficulty level. Therefore, we conclude that the ABT by H.J. Eysenck (1967) cannot explain our data.

Above, we argued that the SOC task is associated mainly with planning, and, if at all, only to a small degree with inhibition. This distinction is important, because ACT predicts that neuroticism affects inhibition, but not planning. In line with these proposals, we found that neuroticism indeed did not impair performance in the SOC task. However, Miyake et al. (2000) suggested that the TOH task, which is related to the SOC task, is associated with inhibition. This seeming contradiction might be explained by the suggestion made by Ozonoff (2004), i.e. that the TOH demands inhibition more than the SOC, mainly because the TOH includes more rules and procedures compared to the SOC [42]. For instance, while the TOH and SOC both require copying a pattern with a certain number of moves [42], only the TOH task is based on differently sized discs which form a pyramidal shape and incorporates an additional rule about the disc sizes (i.e. a larger disc can never be placed on a smaller disc) [42]. Therefore, to resolve rule conflict, part of solving the task is to supress one rule (e.g., selection of bigger discs) and to follow another rule (selection from smaller set of discs). In other words, successful strategies to solve the TOH task require a logical detour which involves inhibition. Therefore, our finding that neuroticism did not affect performance in the SOC task is in accordance with previous literature.

The second aim of the current study was to investigate whether the specific ACT predictions about the affected CES functions can be confirmed. In detail, ACT proposes that high neuroticism considerably impairs switching and inhibition functions rather than storage systems and further CES functions such as planning [19,49]. Therefore, we chose the IED task which is known to heavily rely on set shifting. It has been proposed that inhibition is an essential demand required for set shifting [50]. In more detail, when a shift to a new rule takes place, the old rule needs to be actively inhibited. Consequently, problems with inhibition often result in increased perseveration behaviour, i.e. participants continue to use the old rule [51]. The results clearly demonstrated that the high neurotics made more errors both in the extra dimensional shifts (EDS errors) and total errors. Also, they needed more trials to learn the tasks than the low neurotics, and they completed fewer stages successfully. Similar patterns of these results have been previously reported in high compared to low neurotics in the performance of tasks which have been strongly associated with switching and inhibition (e.g. n-back tasks [30,34], WCST [47]). Taken together, our findings support ACT in showing that switching and inhibition are negatively affected by high levels of neuroticism.

Our conclusions are partially based on assuming that there is no difference between high and low neurotics regarding SOC and SWM performance. While this can be questioned from a statistical point of view, following Cortina & Fkolger, 1998, we believe that our conclusions are warranted. First, one of the main points raised in the context of not finding a difference between two groups is a potential lack of power. However, we would like to point out that the IED task revealed statistically highly significant differences between high and low neurotics. Thus, in principle our design is powerful enough to detect differences in the IED task, which is overall rather similar to the SOC and SWM tasks [52]. Second, assuming that with a larger sample a statistically significant difference between high and low neurotics could be shown in the SOC and SWM as well, it is very likely that this difference will be much smaller. In other words, the effect size is considerably larger for the IED than for the SOC and SWM tasks. This could be explained by the fact that these tasks never rely on only exactly one or two CES sub-functions, but also, to a smaller degree, on a variety of further functions. As an example, the SOC and SWM tasks are probably also demanding inhibition, for instance in the SWM task to help avoiding visiting already visited boxes, but most likely to a much lesser degree than the IED task. For our arguments, it is not vital that there are truly zero differences between groups. Instead, the same conclusions can be drawn based on the assumption that the effects of neuroticism are much stronger in the IED as compared to the SWM and SOC tasks.

It could be argued that the tasks used in the current study did not rely on only one single function of the CES. As noted in the introduction, there is no WM task that measures only a single WM function, because most tasks are tapping into multiple WM functions at the same time [14,15]. However, most WM task will involve a certain functions more than others. Therefore, in the current study, each task predominately measures a certain WM function [14,15]. In addition, our interpretations regarding current results are consistent with previous studies which employed dual tasks and other single tasks [47,53]. For instance, in a series of dual task studies, task demand increased from single tasks (i.e. participants had to respond either to a simple visual or an auditory task) to fixed dual tasks (participants had to respond to both a visual and an auditory task or vice versa, with a fixed order) and then random dual tasks (participants had to respond to a visual and an auditory task, with a random order) [53]. It has been indicated that such manipulation in dual tasks strongly associates with demand on switching and inhibition functions [54,55]. In this context, the aim was to test the effect of neuroticism on switching and inhibition functions [53]. The results showed that high neurotic participants dramatically became slower as compared to low neurotics as task demand increased from single to fixed dual task and from fixed to random dual tasks [53]. It was therefore concluded that as demand increased on switching and inhibition function, the detrimental effect of neuroticism increased [53]. Thus, our interpretation regarding current results seem to be reliable, as such effect of neuroticism has been shown in various types of WM tasks.

To summarize, we propose that the assumption of H.J. Eysenck (1967), i.e. neuroticism impairs the performance of difficult tasks, cannot be generalized to all difficult tasks. As ACT proposed, it seems neuroticism instead impairs performance when the tasks are associated specifically with the switching and inhibition functions of the central executive system, but not with the planning function [19,49,56]. While our research has shown that the impairments are not generalizable to all CES functions (because planning was not affected), future studies need to test the predictions of the ACT further. For instance, ACT proposes that the CES function of updating should also be affected, which we did not test in the current study. Also, further functions have been assigned to the CES, such as monitoring, for which it is currently unknown whether they are affected by neuroticism.

In conclusion, the present results demonstrate that highly neurotic participants showed impaired performance in switching and inhibition, but not in spatial working memory. The latter was observed even in difficult task conditions, illustrating that neuroticism does not affect all kinds of difficult tasks. Understanding the detailed effects of neuroticism on CES functioning will bring us closer to a more comprehensive conceptualization of the cognitive impairments in high neurotics. The current study has been conducted in university students therefore the current result could be considered as sample specific, it would be useful if future investigations replicate this study in other healthy samples apart from university students. In addition, in the current study we aimed at resolving the conflict between assumption of arousal based theory [4] and attentional control theory [19] which makes quite specific assumptions. Future studies could consider other theoretical accounts as well in relation to effect on neuroticism on the cognitive system.

## Supporting information

S1 File Dataset. (SAV)
